# A Communal Bacterial Adhesin Anchors Biofilm and Bystander Cells to Surfaces

**DOI:** 10.1371/journal.ppat.1002210

**Published:** 2011-08-25

**Authors:** Cedric Absalon, Katrina Van Dellen, Paula I. Watnick

**Affiliations:** Division of Infectious Disease, Children's Hospital, Boston, Massachusetts, United States of America; Tufts University School of Medicine, United States of America

## Abstract

While the exopolysaccharide component of the biofilm matrix has been intensively studied, much less is known about matrix-associated proteins. To better understand the role of these proteins, we undertook a proteomic analysis of the *V. cholerae* biofilm matrix. Here we show that the two matrix-associated proteins, Bap1 and RbmA, perform distinct roles in the biofilm matrix. RbmA strengthens intercellular attachments. In contrast, Bap1 is concentrated on surfaces where it serves to anchor the biofilm and recruit cells not yet committed to the sessile lifestyle. This is the first example of a biofilm-derived, communally synthesized conditioning film that stabilizes the association of multilayer biofilms with a surface and facilitates recruitment of planktonic bystanders to the substratum. These studies define a novel paradigm for spatial and functional differentiation of proteins in the biofilm matrix and provide evidence for bacterial cooperation in maintenance and expansion of the multilayer biofilm.

## Author Summary

The bacterial multilayer biofilm consists of matrix-enclosed cells attached to each other to form large aggregates. The base of these aggregates may be attached to a living or non-living surface. The biofilm matrix most often contains at least one exopolysaccharide component and may also contain protein and DNA. While much is known about the exopolysaccharide component of the Gram-negative biofilm matrix, little is known about the function of biofilm matrix proteins. We hypothesized that the biofilm matrix might harbor proteins with diverse functions. Therefore, we undertook the first proteomic analysis of the biofilm matrix of a Gram-negative bacterium, *V. cholerae*. We subsequently focused on Bap1 and RbmA, two proteins that are abundant in the biofilm matrix. RbmA, which strengthens intercellular interactions, was found to be evenly distributed in the biofilm. In contrast, communally synthesized Bap1 was concentrated at the biofilm-surface interface and stabilized the association of the multilayer biofilm with the surface. Furthermore, the addition of purified Bap1 increased attachment of free-swimming cells to a surface. These studies provide evidence for spatial and functional differentiation of proteins in the biofilm matrix and suggest bacterial cooperation in stabilization of multilayer biofilm surface association and recruitment of new members.

## Introduction

Bacterial biofilm formation is the process by which bacteria attach to abiotic surfaces, the surfaces of other unicellular organisms, the epithelia of multicellular organisms, and interfaces such as that between air and water. Surface adhesion enables bacteria to arrange themselves favorably in their environment and, therefore, is critical to environmental adaptation and survival.

Surface-attached bacteria may form either a single layered structure, known as a monolayer, or a multilayer biofilm [1]. Bacterial cells join monolayer and multilayer biofilms in response to distinct environmental signals, use distinct structures for adhesion in these two biofilms, and develop distinct transcriptional profiles within these two structures [2], [3]. However, the critical difference between these two types of biofilms is the extracellular matrix that surrounds cells in the multilayer biofilm. This matrix is comprised of biological polymers such as exopolysaccharide, protein, and DNA [4]. The matrix not only mediates bacterial aggregation and surface attachment but may also serve as a reservoir for extracellular, degradative enzymes and the nutrients released by their function. Therefore, the multilayer biofilm affords the bacterium advantages that monolayer biofilm does not.

*Vibrio cholerae* is a halophilic Gram-negative bacterium that causes the severe diarrheal disease cholera. *V. cholerae* makes two types of multilayer biofilms. One is dependent on environmental Ca^2+^ concentrations comparable to those found in seawater, while the other is dependent on the synthesis of an exopolysaccharide termed VPS [2], [5], [6], [7]. The genes required to synthesize VPS are primarily found in two large operons within the VPS island, one of which encodes the proteins VpsA through VpsK and the other of which encodes VpsL through VpsQ [8]. Transcription of these operons is controlled by a complex regulatory network, suggesting that the ability to limit biofilm matrix synthesis to a highly specific environmental niche confers a survival advantage [9], [10], [11], [12], [13], [14], [15], [16], [17].

While most studies suggest that the VPS-dependent *V. cholerae* biofilm is not important for colonization of the human intestine [18], [19], [20], this biofilm may be important for environmental persistence. Surface-attached *V. cholerae* predominate in the environment [21], [22]. Multiple avenues of evidence suggest that the chitinaceous surfaces of arthropods are an important substratum for *V. cholerae* biofilm formation [23], [24], [25], [26], [27]. Furthermore, *V. cholerae* is especially well adapted to life on chitin because of its many chitinolytic enzymes, the marked modulation of its transcriptome by the degradation products of chitin [28], and activation of its natural competence by chitin [28], [29], [30], [31].

Our laboratory and others have identified several environmental signals that activate VPS-dependent *V. cholerae* biofilm formation [2], [5], [12], [32], [33], [34], [35]. Among these are sugars transported by the phosphoenolpyruvate phosphotransferase system or PTS. Chitobiose and N-acetylglucosamine, which are degradation products of chitinaceous surfaces of arthropods, are transported exclusively by the PTS [36], [37]. Therefore, in the aquatic environment, association with arthropods is likely correlated with formation of a VPS-dependent biofilm.

To gain insight into the role of biofilm matrix-associated proteins in *V. cholerae* surface attachment, we set out to define the proteome of the *V. cholerae* biofilm matrix. Here, we present evidence that the biofilm matrix selectively retains secreted proteins. Furthermore, we show that RbmA and Bap1, two proteins of previously unknown function [3], [38], [39], [40], are present in the biofilm matrix. While RbmA functions similarly to previously identified biofilm matrix proteins in that it strengthens intercellular interactions [41], [42], [43], Bap1, which is jointly synthesized by biofilm-associated bacteria, is concentrated at the base of the biofilm where it reinforces the association of the biofilm with the surface and accelerates attachment of bystander bacteria not yet primed for biofilm matrix synthesis. These studies present evidence for specialization of proteins in the bacterial biofilm matrix and for bacterial cooperation in maintaining and expanding surface-associated biofilms.

## Results

### Identification of biofilm matrix-associated proteins

Biofilm matrix proteins were isolated by a variety of methods. Briefly, biofilms were disrupted by vortexing in the presence or absence of 1 mm glass beads. Furthermore, biotinylation of extracellular proteins prior to biofilm disruption and subsequent neutravidin affinity purification were used to enrich for extracellular proteins. The protein mixtures prepared by these methods were analyzed by MS/MS. We then used *in silico* methods (Genome Atlas) to predict the subcellular localization of identified proteins. As shown in Figure S1, the proportion of recovered proteins that were predicted to be extracytoplasmic increased with biotinylation. Gentler methods of biofilm disruption also resulted in isolation of a larger proportion of predicted extracytoplasmic proteins. However, the most gentle disruption methods yielded fewer proteins overall and, therefore, a smaller number of secreted proteins.

The 74 predicted extracytoplasmic proteins identified by these methods are listed in Table S1. Based on either known function or bioinformatics, we predicted that 10 of these proteins were secreted and, therefore, were candidate biofilm matrix-associated proteins (Table 1). In addition, 17 of these proteins were located in the outer membrane (OM), and 26 of these proteins were located in the periplasm. The location of 18 proteins could not be predicted with certainty (Table 2). Citrate synthase (VC2092) and a putative acetyl CoA synthase homolog (VCA0139), which were predicted to have transmembrane domains, are most likely in the inner membrane. No additional inner membrane proteins were identified. NusA (VC0642), a transcription elongation factor we identified in the proteomic analysis, was predicted to be secreted. However, because of its function, we hypothesize that it is cytoplasmic.

**Table 1:** Secreted proteins identified in preparations of biofilm matrix.

Genomic Locus	Annotation
VC0409	MshA
VC0928	RbmA
VC0930	RbmC
VC2142	FlaB
VC2143	FlaD
VC2187	FlaC
VC2188	FlaA
VCA0027	ChiA-2
VCA0219	HlyA
VCA0865	HAP

**Table 2:** Extracytoplasmic proteins of unknown location.

Genomic Locus	Annotation
VC0174	hypothetical
VC0430	immunogenic protein
VC0483	hypothetical
VC1101	hypothetical
VC1154	hypothetical
VC1334	hypothetical
VC1384	hypothetical
VC1523	hypothetical
VC1834	hypothetical
VC1853	hypothetical
VC1887	hypothetical
VC1894	hypothetical
VC2168	hypothetical
VC2517	hypothetical
VCA0026	hypothetical
VCA0058	conserved, hypothetical
VCA0144	immunogenic protein
VCA0900	hypothetical

Secreted proteins identified in our analysis included those forming bacterial appendages such as the mannose-sensitive hemagglutinin type IV pilus (MshA) and the flagellum as well as RbmA and RbmC, two proteins of unknown function that alter biofilm formation and are co-regulated with the VPS synthesis genes [3], [38], [39], [40]. Three proteins not previously associated with biofilms were also identified, namely a hemolysin (HlyA, VCA0219), a chitinase (VCA0027), and the hemagglutinin/protease (HAP; VCA0865).

We hypothesized that cell-associated proteins should be similarly represented in our analyses if they represented residual cellular material contaminating the biofilm matrix preparation. In fact, while 35% of the periplasmic and OM proteins identified were found in three or more biofilm matrix preparations, only 7% of all predicted cytoplasmic proteins were identified in 3 or more preparations. Furthermore, only two putative inner membrane proteins were identified. One possibility is that a common step in the purification process resulted in formation of spheroplasts, releasing outer membrane and periplasmic proteins into the supernatant during the purification process. Another possibility is that these OM and periplasmic proteins signify the presence of outer membrane vesicles in the biofilm matrix.

### Bap1 and RbmC perform redundant functions in *V. cholerae* biofilm formation

**Figure 1:**
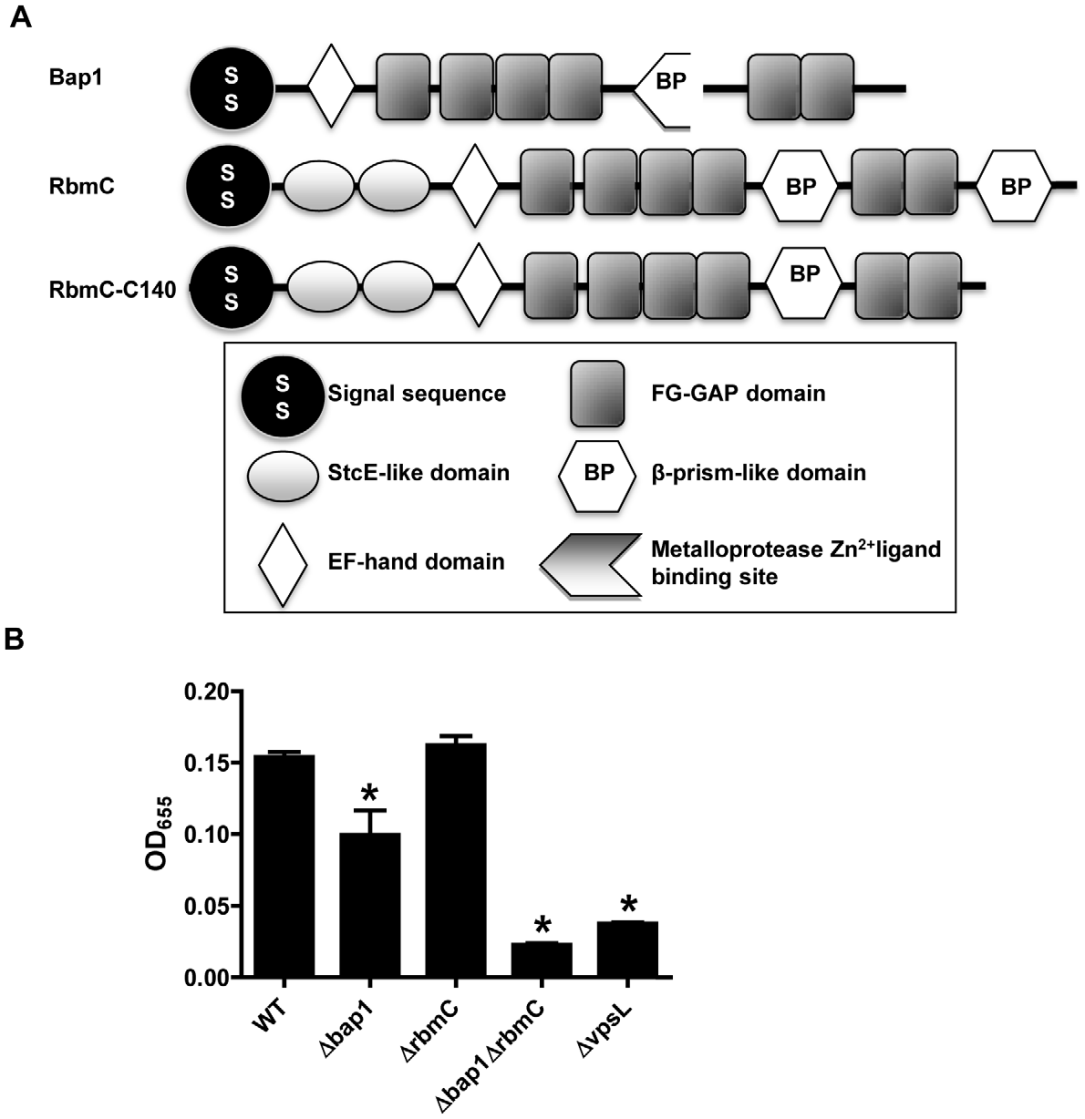
Bap1 and RbmC perform redundant functions in biofilm formation. (A) Domain analysis of Bap1 and RbmC. Bap1 consists of a signal sequence, an EF hand domain, and a β-prism lectin domain surrounded by six FG-GAP domains. RbmC has two additional StcE-like domains at the N-terminus and an additional β-prism domain at the C-terminus. (B) Quantification of biofilms formed by wild-type *V. cholerae* (WT), a Δ*vpsL* mutant, a Δ*bap1* mutant, a Δ*rbmC* mutant, and a Δ*bap1*Δ*rbmC* mutant. * indicate values that are statistically significantly different from wild-type.

RbmC (957 aa), which was identified in our proteomic analysis, and its homolog Bap1 (691 aa) play uncharacterized and redundant roles in the observed colony morphology and biofilm phenotype of rugose *V. cholerae* variants [39]. The central portions of these proteins are 54% identical and 70% similar and include an EF hand domain, which is predicted to bind Ca^2+^, and a β-prism lectin-like domain surrounded by six FG-GAP domains (Figure 1A). RbmC is longer than Bap1 due to two N-terminal domains of unknown function that are also found in the *E. coli* mucinase StcE and a second C-terminal β-prism domain [44], [45].

We first confirmed that these proteins also serve redundant roles in biofilm formation in our *V. cholerae* strain MO10, which has a smooth rather than rugose colony morphology. As shown in Figure 1B, Δ*bap1* and Δ*rbmC* mutants formed a biofilm, while the double mutant did not. The biofilm defect of the Δ*bap1*Δ*rbmC* mutant could be rescued by a plasmid encoding a wild-type allele of either Bap1 or RbmC (Figure S2). An *rbmC* allele with a truncation of the C-terminal β-prism domain not found in Bap1 (RbmC-C140) also rescued the biofilm defect of the Δ*bap1*Δ*rbmC* mutant (Figure S2). These results suggest that, as previously noted for a rugose variant of *V. cholerae*, Bap1 and RbmC perform redundant functions in the *V. cholerae* biofilm. Furthermore, Bap1 represents the minimal protein required to rescue the Δ*bap1*Δ*rbmC* mutant phenotype.

### A subset of candidate biofilm matrix-associated proteins are visualized in the biofilm matrix

**Figure 2:**
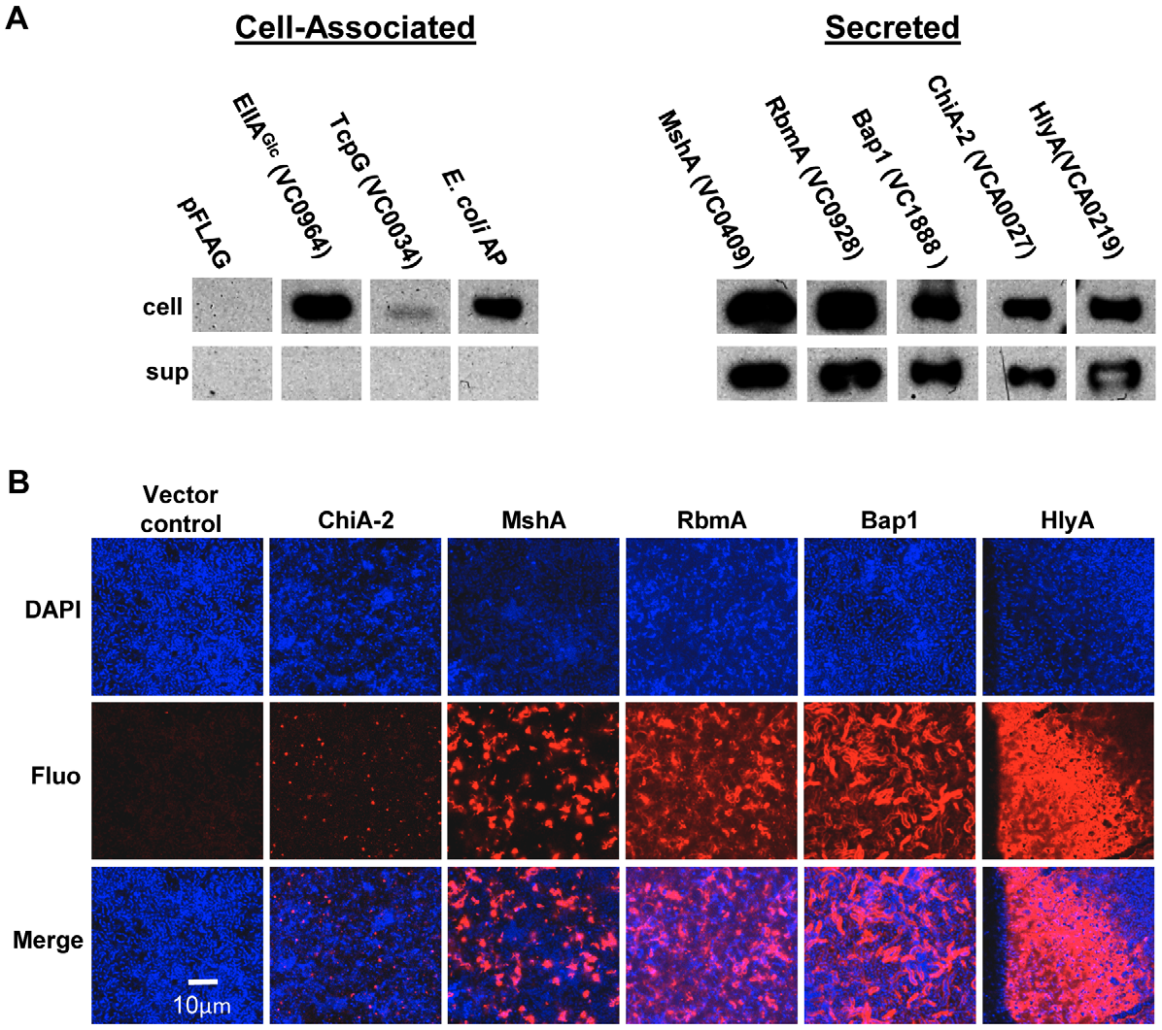
Secreted proteins identified in proteomic analyses are retained in the biofilm matrix. (A) Western blot of cell pellet and supernatant fractions for wild-type *V. cholerae* with an empty vector or a vector encoding a FLAG-tagged protein as labeled. (B) Transverse sections at the level of the substratum through biofilms containing FLAG-tagged proteins as noted. Proteins were visualized by immunofluorescent staining.

Our proteomic analysis identified ten candidate matrix-associated proteins (Table 1). ChiA-2, MshA, Bap1, RbmA, and the hemolysin HlyA were selected for further study. To determine whether these proteins were secreted by *V. cholerae*, the gene encoding each of these proteins was cloned between an inducible promoter and a C-terminal FLAG tag. As negative controls, we also cloned EIIA^Glc^ (VC0964), a cytoplasmic component of the phosphoenolpyruvate phosphotransferase system, as well as *Escherichia coli* alkaline phosphatase (AP) and TcpG (VC0034), two periplasmic proteins. Each of these plasmids was introduced into *V. cholerae*. After culture in LB broth, the cells and supernatant were separated by centrifugation, and the presence of the tagged protein in each fraction was assessed by Western analysis (Figure 2A). The negative controls EllA^Glc^, TcpG, and AP were found in the cell pellet only. The secreted proteins chosen for further study were all found in the supernatant to varying degrees.

To determine if these secreted proteins were retained in the biofilm matrix, we formed biofilms with wild-type *V. cholerae* constitutively expressing affinity-tagged versions of each of these proteins. Biofilms were rinsed, and immunofluorescence was used to visualize the affinity-tagged proteins in the biofilm matrix. No fluorescence was observed for biofilms formed by strains carrying plasmids encoding the proteins EllA^Glc^, TcpG, or AP (data not shown). As expected, the pilus-forming protein MshA was visualized in the biofilm matrix. In addition, RbmA, Bap1, and HlyA were observed in the biofilm matrix. Although comparable amounts of ChiA-2 were secreted, much less was observed in the biofilm matrix (Figure 2B). This suggests that RbmA, Bap1, and HlyA are selectively retained in the biofilm, while ChiA-2 does not associate strongly with the biofilm matrix.

### Bap1 and RbmA have distinct distributions in the biofilm matrix

**Figure 3:**
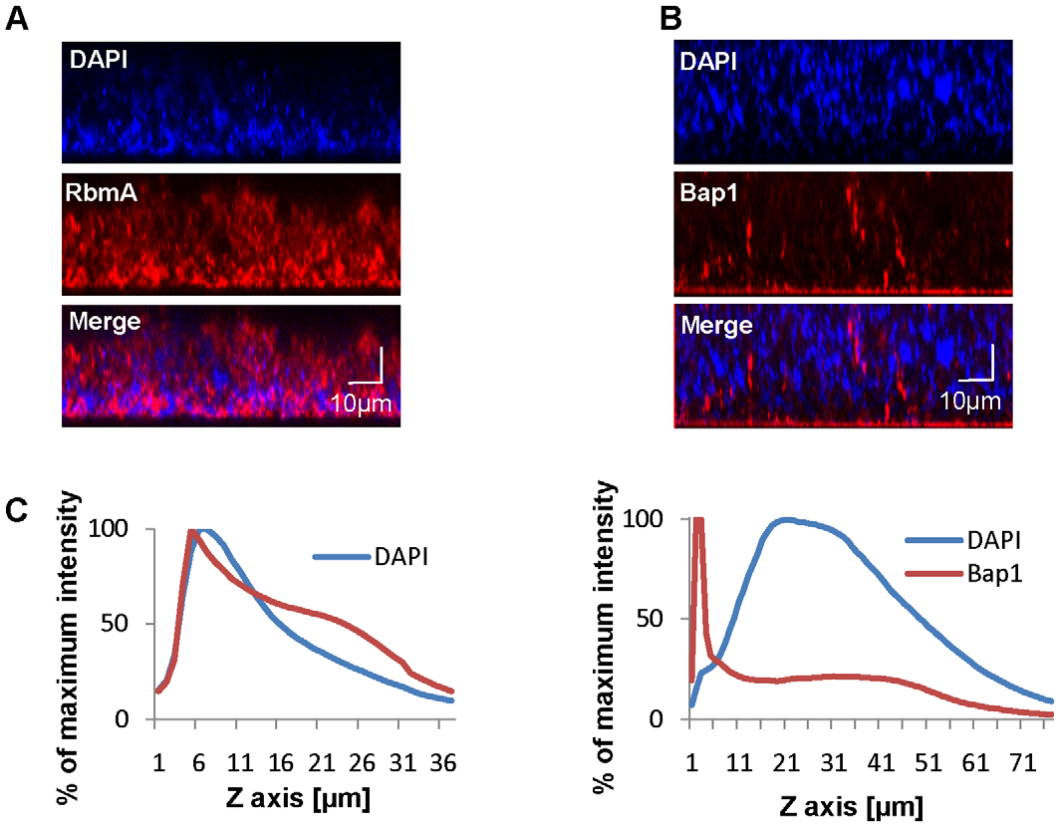
Vertical distribution of Bap1 and RbmA in a native *V. cholerae* biofilm. Immunofluorescent imaging of the vertical distribution of (A) Bap1-FLAG and (B) RbmA-FLAG in a native *V. cholerae* biofilm. Strains harbored a FLAG tag fused to the protein of interest at its chromosomal location. Bacterial DNA was stained with DAPI. (C) Quantification of the fluorescent intensity reflecting Bap1 and RbmA abundance as a function of distance from the substratum.

Bap1 and RbmA were previously found to alter biofilm formation [3], [38], [40]. Therefore, we hypothesized that their role in biofilm formation might be a structural one. To compare the native distributions of Bap1 and RbmA in the biofilm, we fused a FLAG tag to the C-terminal end of Bap1 and RbmA on the chromosome and visualized these tagged proteins in the biofilm by immunofluorescence. As shown in Figure 3A, RbmA was evenly distributed in the vertical dimension, while Bap1 was concentrated at the base of the biofilm. To objectively assess this difference, we measured the total fluorescence intensity in each transverse section. For each biofilm, this measurement was normalized to the transverse section with maximum fluorescence intensity and plotted as a function of distance from the substratum. As shown in Figure 3B, these measurements confirmed that Bap1 was concentrated at the biofilm-surface interface.

**Figure 4:**
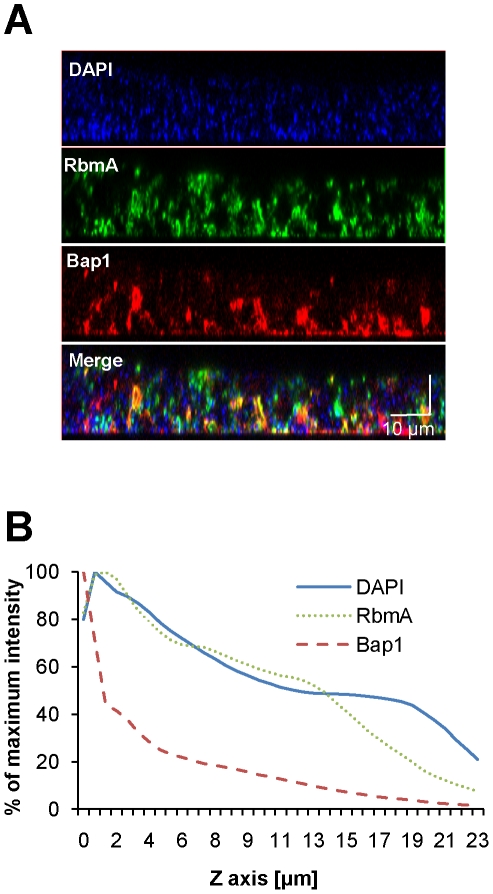
Vertical distribution of constitutively expressed Bap1 and RbmA in the *V. cholerae* biofilm. (A) Vertical section through a biofilm made by co-culturing wild-type *V. cholerae* carrying either a plasmid encoding Bap1-FLAG or a plasmid encoding RbmA-6X-His. Bacterial DNA was stained with DAPI, and Bap1-FLAG and RbmA-6X-His were visualized with FLAG specific and 6X-His specific antibodies, respectively. (B) Quantification of the total fluorescence due to DAPI, Bap1-FLAG or RbmA-6X-His as a function of biofilm height. Fluorescence in each section was normalized to the maximum fluorescence intensity for that biofilm. Vertical sections and quantifications of fluorescence are representative of the three experimental replicates that were performed in parallel.

To determine whether the distinct vertical distributions of Bap1 and RbmA in the biofilm were the result of spatially heterogeneous transcription of *bap1* and *rbmA*, we formed a biofilm with wild-type *V. cholerae* constitutively expressing Bap1-FLAG or RbmA-6XHis from a plasmid. As shown in Figure 4, the vertical distribution of Bap1 and RbmA in these biofilms was similar to that in biofilms expressing Bap1 or RbmA from their respective native promoters. However, with constitutive expression, more Bap1 was observed within the biofilm, most likely due to increased levels of protein. Taken together, our data suggest that the vertical distributions of Bap1 and RbmA in the biofilm are not the result of heterogeneous transcription of *bap1* and *rbmA* within the biofilm. Rather, we hypothesize that Bap1 migrates to the biofilm-substratum interface after secretion from the cell.

**Figure 5:**
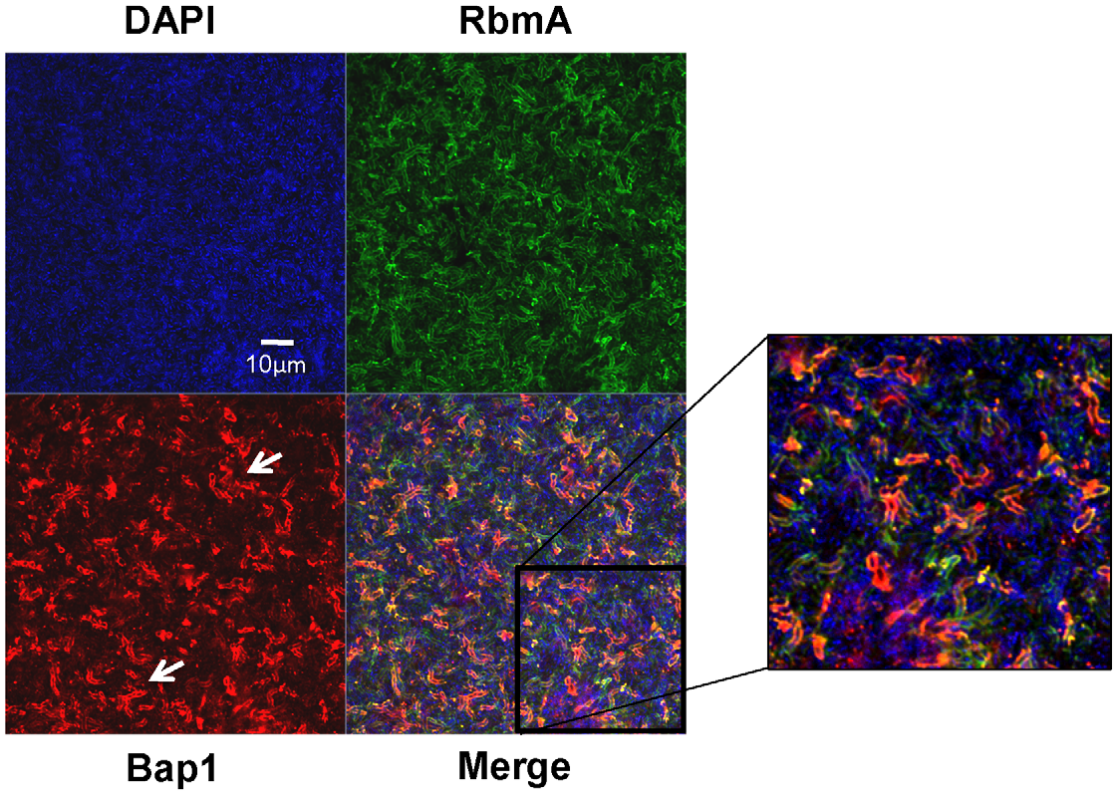
Transverse distribution of constitutively expressed Bap1 and RbmA in the *V. cholerae* biofilm. A transverse section at the level of the substratum of a biofilm made by co-culture of a Δ*bap1* mutant carrying a plasmid encoding Bap1-FLAG and wild-type *V. cholerae* carrying a plasmid encoding RbmA-6X-His. Bacterial DNA was stained with DAPI, and Bap1-FLAG and RbmA-6X-His were visualized by immunofluorescence. Horizontal sections are representative of the three experimental replicates that were performed in parallel. White arrows denote foci of Bap1 staining.

To assess the transverse distribution of Bap1 and RbmA in the biofilm and the extent of co-localization of these two proteins, we combined equal numbers of a Δ*bap1* mutant expressing Bap1-FLAG from a plasmid and wild-type *V. cholerae* expressing RbmA-His from a plasmid. As shown in Figure 5, in transverse sections close to the substratum, Bap1 and RbmA were both distributed around the perimeter of cells, and some co-localization was observed. However, RbmA was more evenly distributed, while foci of increased intensity were observed for Bap1. Similar transverse distributions of each protein were observed in biofilms formed by a *Δbap1* mutant expressing Bap1-FLAG from a plasmid alone and by a *ΔrbmA* mutant expressing RbmA-FLAG from a plasmid alone (data not shown). Based on these observations, we hypothesized that Bap1 might play a different role than RbmA in biofilm formation.

### Exogenously provided RbmA enhances intercellular interactions

**Figure 6:**
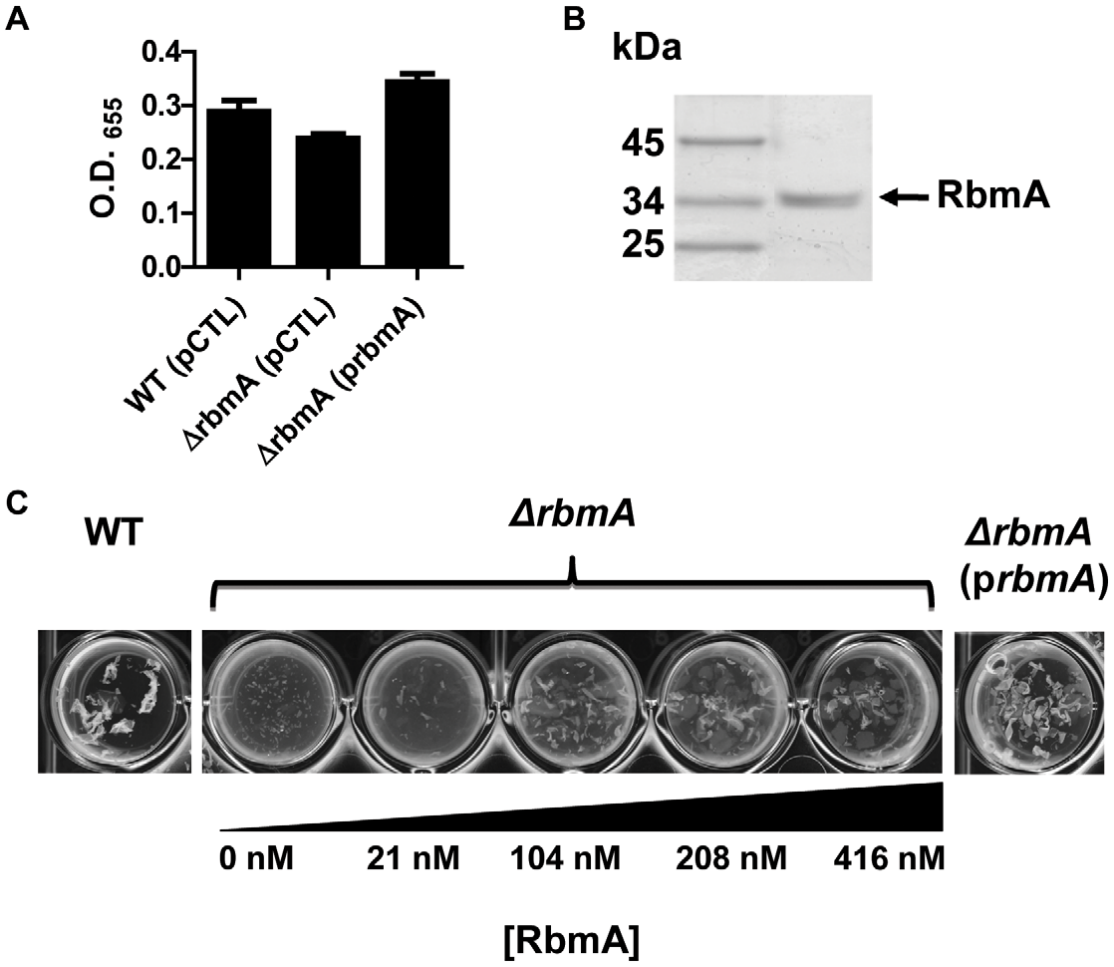
Purified RbmA-FLAG rescues a Δ*rbmA* mutant. (A) Quantification of biofilms formed by wild-type *V. cholerae* or a Δ*rbmA* mutant carrying either an empty vector (pCTL) or a vector encoding a wild-type *rbmA* allele (prbmA). The means and standard deviations were calculated from three experimental replicates. While the biofilms formed by the Δ*rbmA* (pCTL) and the Δ*rbmA* (prbmA) strains were not significantly different from that formed by wild-type *V. cholerae*, the Δ*rbmA*(prbmA) biofilm was significantly greater than the Δ*rbmA*(pCTL) biofilm (p = 0.004). (B) SDS-PAGE analysis of purified RbmA-FLAG. Protein was visualized with Imperial stain. (C) Biofilms formed by a Δ*rbmA* mutant rescued with increasing amounts of purified RbmA-FLAG. Biofilms have been vortexed to illustrate fragmentation of the Δ*rbmA* mutant biofilm.

RbmA alters biofilm stability but not overall biofilm accumulation of rugose variants of *V. cholerae* [38]. In *V. cholerae* O139 strain MO10, we observed that deletion of *rbmA* had a small, statistically insignificant effect on biofilm formation. Rescue of a Δ*rbmA* mutant with a wild-type *rbmA* allele produced a biofilm that was similar to that of wild-type *V. cholerae* but significantly increased as compared with the biofilm of the unrescued mutant (Figure 6A). Vortexing completely dispersed the Δ*rbmA* mutant biofilm, while larger biofilm fragments were observed after similar treatment of the wild-type *V. cholerae* biofilm (Figure 6C). This Δ*rbmA* mutant phenotype could be complemented by expression of a wild-type *rbmA* allele in trans.

We hypothesized that, if secreted RbmA were essential for biofilm integrity, exogenous RbmA should rescue the biofilm defect of a Δ*rbmA* mutant. To test this, we first affinity purified RbmA (Figure 6B). We then allowed the Δ*rbmA* mutant to form a biofilm in the presence of increasing amounts of purified RbmA. Purified RbmA was able to rescue the biofilm defect of the Δ*rbmA* mutant (Figure 6C). We determined that rescue required an RbmA concentration of approximately 416 nM. Assuming all molecules of RbmA are functional, this corresponds to approximately 260,000 molecules per mutant cell.

### Bap1 is involved in surface adhesion

**Figure 7:**
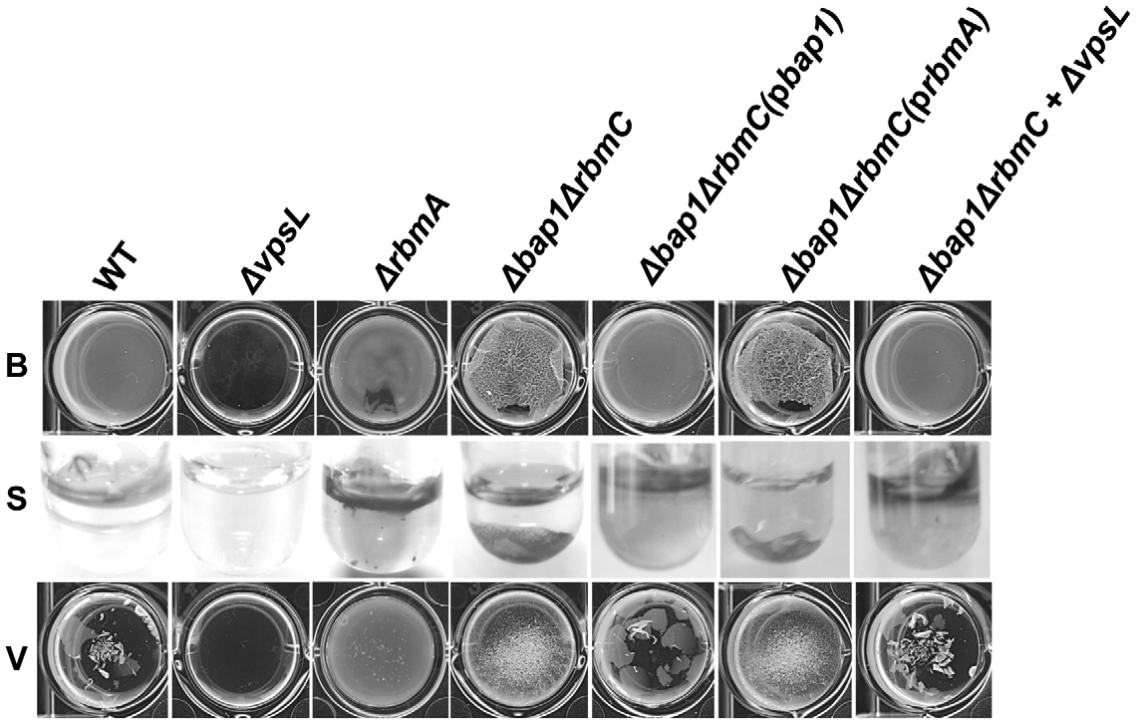
The Δ*bap1*Δ*rbmC* mutant biofilm is loosely adherent to the substratum. Pellicles formed by wild-type *V. cholerae*, a Δ*vpsL* mutant, a *ΔrbmA* mutant, a *Δbap1ΔrbmC* mutant, a Δ*bap1*Δ*rbmC* mutant rescued with Bap1-FLAG or RbmA-FLAG expressed from a plasmid, and a Δ*bap1*Δ*rbmC* mutant co-cultured with a Δ*vpsL* mutant. Pellicles were photographed without agitation (B), after gentle shaking (S), or after vortexing (V).

In a standard assay, the biofilm formed by a Δ*bap1*Δ*rbmC* mutant was indistinguishable from that formed by a Δ*vpsL* mutant (Figure 1B). However, we noticed that, unlike the Δ*vpsL* mutant, the Δ*bap1*Δ*rbmC* mutant formed a pellicle on the liquid surface after 24 hours of static growth. Interestingly, mutation of *bap1* and *rbmC* in a rugose variant of *V. cholerae* was not noted to preserve pellicle formation [39]. One possible explanation for this discrepancy is that, due to a difference in the surface chemistries of smooth and rugose variants, rugose variants do not interact as strongly with the air-water interface in the absence of Bap1 and RbmC. As shown in Figure 7, the pellicle formed by the Δ*bap1*Δ*rbmC* mutant was loosely associated with the glass surface. Gentle shaking dislodged the Δ*bap1*Δ*rbmC* mutant pellicle from the substratum sending it to the bottom of the tube, while the wild-type pellicle remained attached. Furthermore, vortexing of the Δ*bap1*Δ*rbmC* mutant pellicle caused it to fragment into many small pieces. However, these pieces were larger than those observed when a Δ*rbmA* biofilm was vortexed. These defects were rescued by a wild-type *bap1* allele provided in trans but not by *rbmA* (Figure 7), again indicating that Bap1 and RbmA have distinct roles in biofilm formation.

**Figure 8:**
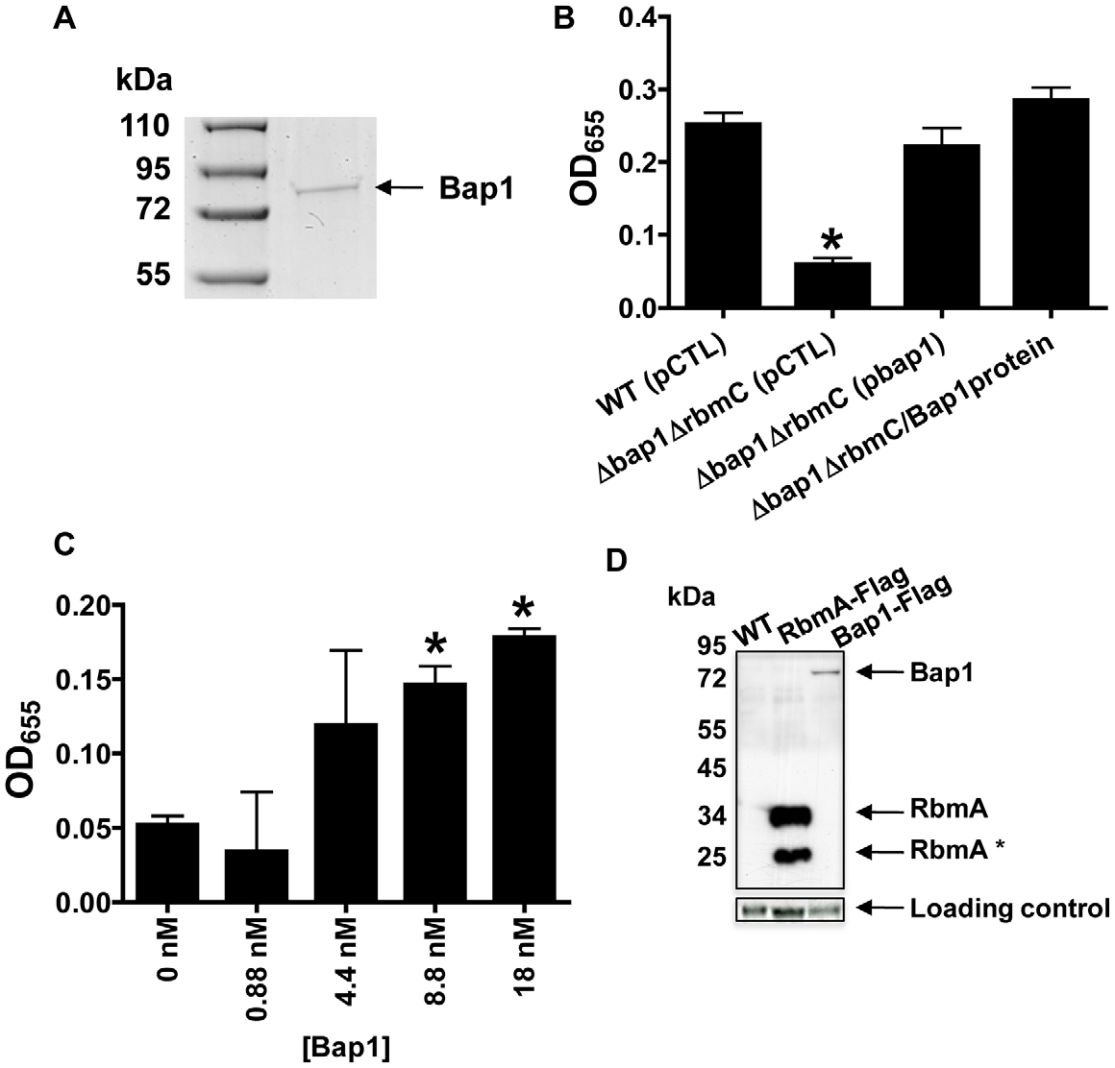
Purified Bap1-FLAG restores biofilm formation to a Δ*bap1*Δ*rbmC* mutant. (A) SDS-PAGE of affinity purified Bap1-FLAG. A single band is seen at the predicted size of 76 kDA. Protein was visualized with Imperial stain. (B) Quantification of biofilms formed by wild-type *V. cholerae* (WT), a Δ *bap1*Δ*rbmC* mutant rescued with either a control plasmid (pCTL) or a plasmid expressing Bap1-FLAG, and Δ *bap1*Δ*rbmC* mutant rescued with purified Bap1 in an 18 nM final concentration. Average measurements and standard deviations were calculated from the results of three experimental replicates. * indicates values that are statistically significantly different from wild-type. (C) Quantification of biofilms made by a Δ *bap1*Δ*rbmC* mutant in the presence of increasing amounts of purified Bap1-FLAG. Average measurements and standard deviations were calculated from the results of three experimental replicates. * indicates values that are statistically significantly different from a biofilm formed by a Δ*bap1*Δ*rbmC* mutant in the absence of purified Bap1. (D) Western analysis of cell extracts prepared from a wild-type biofilm (WT), a biofilm formed with a strain that expresses RbmA-FLAG from the native chromosomal location, and a biofilm formed with a strain that expresses Bap1-FLAG from the native chromosomal location. Blots were probed with an anti-FLAG antibody. Below, the α-subunit of *V. cholerae* RNA polymerase was visualized with an antibody to the *E. coli* protein for use as a loading control. Densitometry showed that approximately 16 times less Bap1 is made in biofilm cells as compared with RbmA.

We hypothesized that if secreted Bap1 were responsible for adhesion of the biofilm to the surface, exogenously provided Bap1 should also rescue the Δ*bap1*Δ*rbmC* mutant biofilm defect. To test this prediction, we used affinity chromatography to purify Bap1-FLAG as shown in Figure 8A. A Δ*bap1*Δ*rbmC* mutant incubated in the presence of purified Bap1 formed a biofilm that was comparable to that of a Δ*bap1*Δ*rbmC* mutant rescued by Bap1 expressed from a plasmid (Figure 8B). To determine the concentration of Bap1 required to restore biofilm formation to the Δ*bap1*Δ*rbmC* mutant, we titrated purified Bap1-FLAG into a Δ*bap1*Δ*rbmC* mutant culture and measured biofilm formation after 24 hours. As shown in Figure 8C, an 8.8 nM solution of Bap1-FLAG was sufficient to restore surface attachment. Assuming all Bap1 molecules are functional, this corresponds to approximately 5,500 Bap1 molecules per bacterial cell. Therefore, approximately 47 times less Bap1 was required than RbmA to form a biofilm with properties similar to that of wild-type *V. cholerae*.

To validate these quantifications in a native biofilm, we used Western analysis to estimate the relative quantities of Bap1-FLAG and RbmA-FLAG synthesized in biofilms formed with *V. cholerae* strains expressing either Bap1-FLAG or RbmA-FLAG from the native chromosomal location (Figure 8D). Two bands were always observed for biofilm-associated RbmA, suggesting that RbmA undergoes proteolysis in the biofilm. Including both RbmA bands in the calculation, we determined that there was approximately 16 times less Bap1 in biofilm preparations as compared with RbmA, recapitulating our results with purified protein. We hypothesize that less Bap1 is required in the biofilm because it principally associates with the base of the biofilm, whereas RbmA is distributed evenly throughout.

### Bap1 is a communal resource, while VPS is not

**Figure 9:**
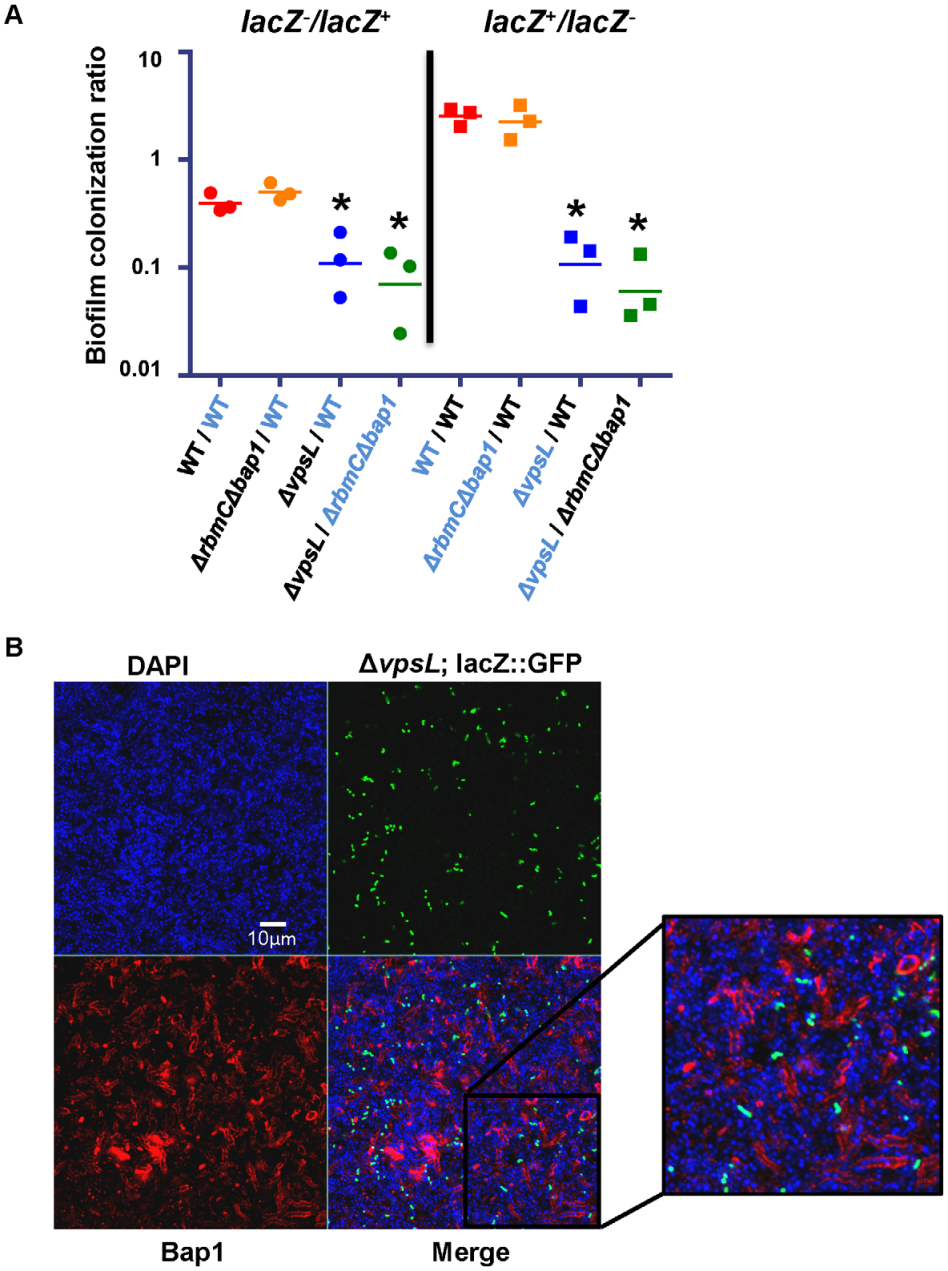
Bap1 is a shared resource; VPS exopolysaccharide is not. (A) Ratio of biofilm-associated CFU for a panel of co-cultured strains including wild-type *V.* cholerae (WT), a Δ*vpsL* mutant, and a Δ *bap1*Δ*rbmC* mutant. Strains were labeled by inactivation of the *lacZ* gene. Label-swapping experiments are shown on right. Black labels indicate a *lacZ* ^−^ strain, while blue labels indicate a *lacZ* ^+^ strain. Three experimental replicates are shown for each condition. * indicates ratios that are statistically significantly different from that calculated for the competition of *lacZ* ^+^ wild-type *V. cholerae* against *lacZ* ^−^ wild-type *V. cholerae*. (B) Transverse section at the level of the substratum through a biofilm formed by co-culture of a Δ*bap1*Δ*rbmC* mutant with a Δ*vpsL* mutant carrying chromosomally encoded GFP and a plasmid encoding Bap1-FLAG. The biofilm is comprised primarily of Δ*bap1*Δ*rbmC* mutant cells surrounded by Bap1-FLAG donated by the *ΔvpsL* mutant. *ΔvpsL* mutants are excluded from the biofilm.

Because exogenously provided Bap1 restored biofilm surface adhesion to a Δ*bap1*Δ*rbmC* mutant, we questioned whether Bap1 synthesis could be a joint venture in the biofilm community. To test this, we co-cultured a Δ*bap1*Δ*rbmC* mutant with a Δ*vpsL* mutant. As shown in Figure 7, this produced a biofilm that was comparable to that of wild-type *V. cholerae.* We rationalized that (i) this biofilm might be comprised chiefly of Δ*vpsL* mutant cells because the Δ*bap1*Δ*rbmC* mutant was providing it with the requisite biofilm exopolysaccharide, (ii) the Δ*bap1*Δ*rbmC* mutant might predominate because the Δ*vpsL* mutant was providing it with the requisite Bap1 and/or RbmC, or (iii) approximately equal numbers of these two mutants might be found in the biofilm because each was providing the other with the requisite materials for biofilm formation. To determine whether Bap1, VPS, or both were shared resources within the biofilm, we performed a series of co-culture biofilm experiments using *lacZ* as a marker and determined the relative amounts of each species in the biofilm by plating on media containing 5-bromo-4-chloro-3-indolyl-β-D-galactopyranoside (X-Gal). As shown in Figure 9A, although the *lacZ* ^+^ strain always had a slight advantage in the biofilm, cells lacking Bap1 and RbmC were always found in the biofilm when co-cultured with cells that were able to produce these proteins. In contrast, cells that were unable to synthesize VPS were always excluded from the biofilm in spite of co-culture with cells that were able to synthesize VPS. Based on these findings, we hypothesize that Bap1 is a shared biofilm resource, but VPS is not.

To document communal Bap1 in the *V. cholerae* biofilm, we then co-cultured a Δ*bap1*Δ*rbmC* mutant with a Δ*vpsL* mutant expressing GFP from a chromosomal location and Bap1-FLAG from a plasmid. The biofilms harvested from these co-culture experiments were visualized by microscopy after immunofluorescent staining of Bap1-FLAG and DAPI staining of bacterial DNA. As expected, approximately one GFP-labeled Δ*vpsL* mutant cell was observed in the biofilm for every GFP-negative Δ*bap1*Δ*rbmC* mutant cell (Figure 9B). However, the perimeter of many Δ*bap1*Δ*rbmC* mutant cells exhibited Bap1-FLAG-based immunofluorescence. To confirm that this observation was not the result of transfer of the plasmid from the Δ*vpsL* mutant to the Δ*bap1*Δ*rbmC* mutant, we documented that all Δ*bap1*Δ*rbmC* mutant cells in the biofilm remained sensitive to ampicillin (data not shown).

Our results confirm that Bap1-FLAG provided by a Δ*vpsL* mutant can be incorporated into the Δ*bap1*Δ*rbmC* mutant biofilm. These findings indicate that Bap1 is a communal resource. In contrast, because Δ*vpsL* mutant cells were excluded from both Δ*bap1*Δ*rbmC* mutant and wild-type *V. cholerae* biofilms, we conclude that VPS produced by neighboring cells is not available to the Δ*vpsL* mutant and, therefore, that unlike Bap1, the biofilm exopolysaccharide VPS is not a communal resource but instead tightly associated with the cell of origin.

### Bap1 mediates adhesion of bystander cells

**Figure 10:**
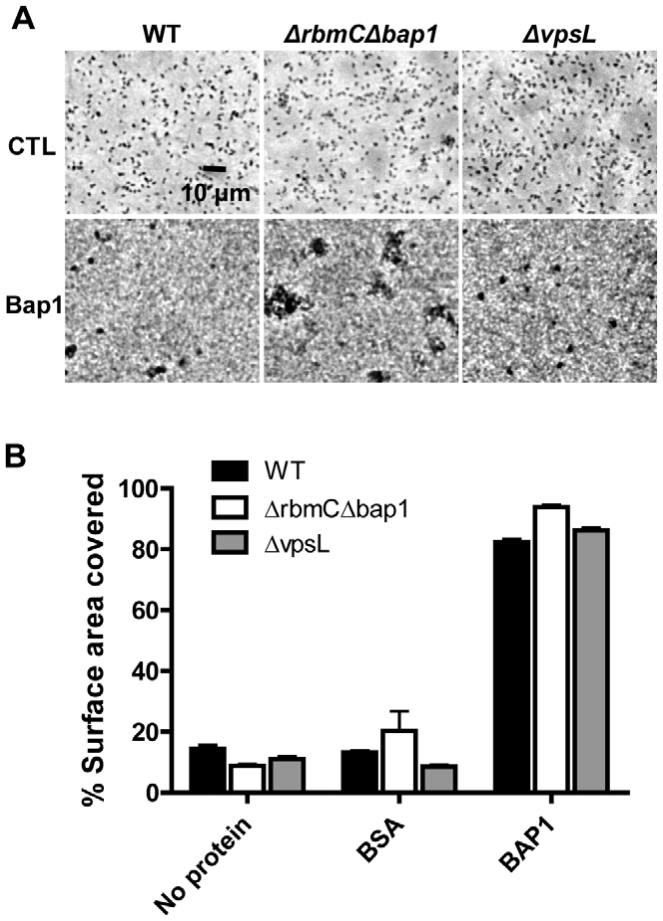
Purified Bap1-FLAG can mediate surface adhesion in the absence of VPS. (A) Surface adhesion by wild-type *V. cholerae*, a Δ *bap1*Δ*rbmC* mutant, or a Δ*vpsL* mutant in monolayer minimal medium either alone (no protein), supplemented with purified Bap1-FLAG protein (Bap1), or supplemented with BSA. Phase contrast microscopy was used to obtain images. (B) Surface area coverage of monolayers illustrated in (A). Average measurements and standard deviations were calculated from six microscope fields derived from three experimental replicates.

We questioned whether Bap1 could also increase surface adhesion of bystander cells not yet committed to the sessile life style, as this would have implications for the role of Bap1 in biofilm expansion. We previously identified a medium in which *V. cholerae* does not synthesize enough of the biofilm matrix components to proceed past the monolayer stage of biofilm development [2]. We cultured wild-type *V. cholerae*, a Δ*bap1*Δ*rbmC* mutant, or a Δ*vpsL* mutant in monolayer minimal medium with supplemented with purified Bap1. As shown in Figure 10A and quantified in Figure 10B, Bap1 increased surface adhesion of wild-type *V. cholerae*, a Δ*bap1*Δ*rbmC* mutant, and a Δ*vpsL* mutant in monolayer minimal medium, while a control protein, bovine serum albumin (BSA), had no effect. This suggests that communal Bap1 secreted by nearby biofilm cells may also increase surface adhesion of bystanders that have not yet been reprogrammed for biofilm matrix synthesis.

## Discussion

The exopolysaccharide component of the bacterial biofilm matrix has been studied intensively [8], [46], [47], [48], [49], [50], [51]. More recently, components such as DNA and protein have been identified in the matrices of some bacterial biofilms. Here we provide the first proteomic analysis of a Gram-negative biofilm matrix. Our analysis revealed 10 secreted proteins, 43 periplasmic and outer membrane proteins, and 18 putative extracytoplasmic proteins whose location could not be predicted.

OM and periplasmic proteins were much more likely to be identified in multiple matrix preparations than inner membrane and cytoplasmic proteins, suggesting that these proteins may not be artifacts caused by cell lysis but rather the contents of biofilm-associated OM vesicles. Outer membrane vesicles are retained in the biofilms of *Pseudomonas aeruginosa* and *Helicobacter pylori*, and these vesicles appear to play a role in biofilm formation [52], [53], [54]. Furthermore, there is evidence that the compositions of membrane vesicles derived from the biofilm and from culture supernatants are distinct [52], [55]. *V. cholerae* has been reported to release outer membrane vesicles [56], [57], [58]. However, additional investigations are required to confirm the presence of these vesicles in the biofilm matrix and to determine their role in biofilm formation.

We studied four secreted proteins identified in our preliminary analysis in addition to MshA. The chitinase, ChiA-2, showed minimal retention in the biofilm matrix. However, three proteins of unknown function, Bap1, RbmA, and HlyA showed extensive association with the matrix. RbmA has no conserved domains of known function. Bap1, its homolog RbmC, and HlyA, which all contain at least one β-prism lectin domain, form a paralogous family in *V. cholerae*. We hypothesize that these secreted proteins are selectively retained in the biofilm, perhaps by binding to specific moieties in the polysaccharide scaffold.

Bap1 and RbmA were previously shown to play an undefined role in *V. cholerae* biofilm formation [3], [38], [39]. Here we show that RbmA and Bap1 have distinct distributions in the biofilm matrix. RbmA surrounds biofilm-associated cells throughout the biofilm and reinforces intercellular contacts from this location. In contrast, Bap1 concentrates around cells that form the biofilm-surface interface and stabilizes adhesion of the biofilm to surfaces. The distinct distribution of these proteins is not the result of heterogeneous expression within the biofilm. Rather, we hypothesize that it is the result of self-segregation after secretion from the cell. This is the first example of spatial and functional differentiation of secreted structural proteins in a Gram-negative biofilm matrix.

Biofilm matrix polysaccharide is considered to be a jointly synthesized, shared resource. We show here that this is not the case for the *V. cholerae* biofilm matrix. While the biofilm matrix protein Bap1 is a communal resource, VPS benefits only cells from which it is synthesized. Therefore, the *V. cholerae* biofilm exopolysaccharide is not freely secreted and available to the entire community.

Lastly, our results suggest that matrix-associated proteins may play an important role in expansion of existing bacterial biofilms on surfaces. Exogenous Bap1 increases surface adhesion of planktonic bystanders as well. Because nutritional signals and surface attachment are strong activators of the biofilm matrix synthesis genes, in aquatic environments, it is unlikely that planktonic Bap1 and RbmC would be synthesized by planktonic cells in quantities sufficient to increase surface attachment. Rather, we envision that Bap1 and RbmC secreted from an existing biofilm would condition surrounding surfaces, increasing the probability of bystander cell attachment.

These studies reveal a new paradigm for the bacterial biofilm matrix in which the biofilm exopolysaccharide forms a cell-associated scaffold to which communal biofilm matrix proteins adhere, possibly through carbohydrate-binding domains. These proteins may fulfill specialized structural roles or enable cooperative augmentation of the biofilm.

## Materials and Methods

### Bacterial strains, plasmids, and media

The bacterial strains and plasmids used in this study are listed in Table S2. Vectors used for protein expression included either an IPTG inducible promoter and a FLAG-tag (pFLAG-CTC, Sigma-Aldrich) or an arabinose inducible promoter and a 6X-His tag (pBAD-Topo, Invitrogen). Bacteria were cultivated either in Luria-Bertani broth (LB) or monolayer minimal media [2]. Where indicated, streptomycin (100 µg/ml), ampicillin (50 or 100 µg/ml), arabinose (0.04% wt/vol), and Isopropyl β-D-1-thiogalactopyranoside (1 mM) (IPTG) were added to the growth medium. A 0.1 M phosphate-buffered saline solution (PBS) (pH 7.0) was used in initial biofilm washes, and a 0.1 M Tris-buffered saline solution (TBS) (ph 7.0) was used to wash biofilms after biotin labeling.

### Identification of biofilm matrix-associated proteins

10 mls of LB broth supplemented with streptomycin was added to a Petri dish and inoculated with *V. cholerae*. A biofilm including a pellicle formed over 48 hours of static incubation at 27°C. After incubation, the associated planktonic cells were removed. The remaining biofilm was washed by addition of PBS, agitation on a rotary shaker for 5 minutes with PBS, removal of PBS and non-attached cells, and addition of fresh PBS. This procedure was repeated twice. Matrix proteins were then prepared using each of the following four protocols (Figure S1). In preparation (i), the biofilm was disrupted in the presence of 1.0 mm glass beads (Biospec) and centrifuged to remove particulates. For preparations (ii), (iii), and (iv), a cell surface biotinylation kit (Pierce) was used to biotinylate extracytoplasmic proteins in the washed biofilm according to the manufacturer's instructions. After biotinylation, the biofilm was transferred to a 50 ml conical tube containing 2 mls of PBS. Disruption of the pellicle was carried out by ten sonication cycles of 10 sec (iii) or by vortexing in the presence (ii) or absence (iv) of 1.0 mm glass beads (Biospec) for one minute. The mixtures were then centrifuged at 20, 000× g for 30 min in the cold to remove particulates, the supernatants were applied to Neutravidin-agarose resin (Pierce), and the resin was washed several times with PBS. Biotinylated proteins were eluted from the resin by incubation with PBS to which 50 mM DTT had been added. This disrupts the disulfide bonds bridging biotin residues to extracellular proteins.

The four mixtures of proteins were precipitated with trichloroacetic acid, resuspended in SDS-PAGE loading buffer, run into a 4–20% gradient SDS-polyacrylamide gel, and then sent to the Taplin Mass Spectrometry Facility where the gel was cut into pieces and subjected to an in-gel trypsin digestion procedure. Peptides were extracted from the gel, dried in a speed-vac, and reconstituted in 5–10 µl of HPLC solvent A (2.5% acetonitrile, 0.1% formic acid). Each sample was loaded via a Famos auto sampler (LC Packings) onto a nano-scale reverse-phase HPLC capillary. Eluted peptides were subjected to electrospray ionization and then entered into an LTQ linear ion-trap mass spectrometer (ThermoFisher). Peptide sequences were determined by matching protein databases with the acquired fragmentation pattern by the software program, Sequest (ThermoFisher).

### Protein overexpression

The ORFs of interest were amplified by PCR using primers including the start and stop codons of each gene of interest. For cloning into pBAD-Topo, PCR products were inserted into the expression vector according to the manufacturer's protocol (Invitrogen). For cloning into pFLAG-CTC, either NdeI and KpnI or NdeI and EcoRI restriction sites were included in the PCR primer pairs. The PCR products were then digested and ligated into the expression vector. The ligation products were transformed into *E. coli* TOP10 competent cells and selected on LB agar plates supplemented with ampicillin (100 µg/ml). The presence of the correct insert was confirmed by colony PCR and sequence analysis. Confirmed plasmids were electroporated into *V. cholerae*. *V. cholerae* strains harbouring a pBAD-Topo plasmid were grown in 0.02% arabinose

### Generation of *V. cholerae* strains encoding FLAG-tagged Bap1 and RbmA on the chromosome

C-terminal fragments of *bap1* and *rbmA* were amplified from the p*FLAG-bap1* and p*FLAG-rbmA* plasmids, respectively, by the polymerase chain reaction with the following primers: Bap1 A: ATCGTCTAGAGTGTACGCGGGTTACTACGC and B: GACTGCATGCCAGACCGCTTCTGCGTTCTG and RbmA: A: AGTCTCTAGAGCCAGTGATTGAAGCAAATC and B: GACTGCATGCCAGACCGCTTCTGCGTTCTG. The resulting PCR products were digested with XbaI and SphI and ligated into the multiple cloning site of the suicide plasmid pGP704, and the sequence was confirmed. This plasmid was then integrated into the chromosome by single homologous recombination as previously described [37].

### Mutant construction

The Δ*rbmC* in-frame deletion mutant was constructed as previously described [12]. Briefly, the following primer pairs: Pair 1 A: TGGCGCCATATTCTATGACA and B: TTACGAGCGGCCGCATACACCCTTCGGCTTCATTC and Pair 2 A: TGCGGCCGCTCGTAATATTGGGCTCAACCCACTATG and B: GGCAGTTTAATGGCGATCAT were used to amplify two genome sequences spanning an in-frame deletion in the gene of interest. These DNA fragments were joined by the SOE technique [59], cloned into pCR2.1-TOPO and then subcloned into the suicide vector pWM91 by ligation after digestion with *XhoI* and *SpeI*. This suicide plasmid was used to generate an in-frame deletion in *rbmC* by double homologous recombination [12]. A similar procedure was used for generation of the Δ*rbmA* in-frame deletion mutant using the following primer pairs: Pair 1 A: CGTACTCGAGCACCCACAATTAGTGATCGCT and B: TAACGAGCGGCCGCACAACCATTTGTTTTTACAACTGG and Pair 2 A: TGCGGCCGCTCGTTATAAATTTACCTAGTCACTTAGTCGT and B: TCGACACTAGTCAAACTCTAGAACGGAACAAAA.

### Biofilm assays

Biofilm quantification assays were performed as described previously with the following modifications [13]. Briefly, a single colony of *V. cholerae* was inoculated into 1 ml of LB broth and allowed to grow to mid exponential phase. The culture was then diluted in LB broth to yield an OD_655_ of 0.05 and divided into three disposable glass culture tubes (10 mm ×75 mm). These tubes were incubated statically at 27°C. After 24 hrs, planktonic cells were removed, and the OD_655_ of the cells was measured. Remaining biofilms were washed with PBS and then disrupted by vortexing in the presence of 1 mm beads. The OD_655_ of the resulting cell suspension was measured. For assays of biofilm integrity, biofilms were formed as described above and then either gently shaken or vortexed. All assays were performed in triplicate and statistical significance was determined by a student's t-test.

### Western blot analysis

To evaluate protein secretion, *V. cholerae* was inoculated into 2 mls of LB broth supplemented with IPTG and ampicillin and grown for 6 hours at 37°C with shaking at 200 rpm. The OD_655_ of the final culture was measured, and then the cells were centrifuged at 4°C for 15 minutes at 4500 rpm. The supernatants and cell pellets were separated. Cell pellets were resuspended in the volume of PBS required to yield a final OD_655_ of 1. Five µl of this cell suspension were diluted in 20 µl 1x Laemmli buffer solution and boiled for 5 min. Supernatants were collected and filtered through a 0.25 µm filter. 10 µl of the supernatants were added to 2 µl 5x Laemmli buffer and boiled for 5 min. The protein mixtures in the cell pellets and supernatants were separated by electrophoresis on a 4–20 % precast SDS-PAGE gel (Pierce) and transferred onto a PVDF membrane (Millipore) with a semi-dry transfer apparatus using the Fast Semi-Dry Transfer Buffer (Pierce). The affinity tagged proteins were visualized as follows. Membranes were incubated overnight in a blocking solution consisting of PBS with 0.05% Tween 20 (PBS-T) and 5% skim milk-PBS. The membranes were then incubated with a 1∶10,000 dilution of Anti-FLAG M2-Peroxidase antibody in PBS-T for 1 hour on a rotary shaker. Membranes were washed once for 15 minutes and twice for 5 minutes in PBS-T and then developed using the ECL Plus Western Blotting Detection Reagent (GE Healthcare) according to the manufacturer's instructions.

To evaluate Bap1 and RbmA in the biofilm at native levels, a similar protocol was used with the following modifications: strains carrying either Bap1-FLAG or RbmA-FLAG on the chromosome were allowed to form biofilms for 24 hours in 2 ml of LB. After removal of planktonic cells and spent medium, biofilms were washed with PBS and resuspended in 500 µl of PBS. Biofilm cell extracts were prepared by sonication, and the protein concentrations of the extracts were determined by Bradford assay. 20 µg of each extract was diluted in 20 µL of MilliQ water and 5X Laemmli buffer and separated by SDS-PAGE. As a loading control, the RNA polymerase α-subunit was detected with an antibody raised against the α-subunit of *E. coli* RNA polymerase (Neoclone). Relative amounts of Bap1 and RbmA in the gel were approximated by densitometry analysis using ImageQuant 5.2 (Molecular Dynamics).

### Immunofluorescence

Wells of a 12 well microtiter dish were filled with 2 mls of LB broth supplemented with ampicillin and arabinose, where noted, and a tilted 18 mm ×18 mm glass cover slip was placed in each well. After 24 hours of static culture, the cover slips were placed in 6 well microtiter dishes and washed twice for 5 minutes with 2 mls of PBS on a rotary shaker. The cover slips were then incubated on a rotary shaker for one hour in a blocking solution consisting of PBS supplemented with 3% BSA. This solution was replaced with blocking solution containing Anti-6X His (1∶1, 000 dilution) (Abcam) and/or Anti-FLAG M2 (1∶1, 000 dilution) (Sigma-Aldrich), and the coverslips were then incubated for an additional hour. After this incubation, the cover slips were washed with PBS three times for 5 minutes each time. For labeling of FLAG-tagged proteins with DyLight549, biofilms exposed to the unlabeled Anti-FLAG M2 antibody underwent an additional 45 min incubation with DyLight 549 AffiniPure Rabbit Anti-Mouse IgG H+L (1∶500 dilution) (Jackson ImmunoResearch). For His-tagged proteins, the same procedure was used with an Alexa Fluor 488 Goat Anti-Rabbit Antibody (Invitrogen). The cover slips were then washed in PBS three times, for 5 minutes each time. Where indicated, the cover slips were also incubated with a 1 mg/ml DAPI solution for 5 min. Cover slips were mounted on concave glass slides filled with PBS and then sealed with nail polish. Confocal images were acquired at the Children's Hospital, Boston Imaging Core with a LSM700 microscope (Zeiss) equipped with a 63X objective and 405, 488, and 555 nm laser lines. A computer equipped with ZEN 2009 software was used to acquire and process images. As a control, a DAPI-stained biofilm was imaged before and after immunofluorescence manipulations. Very little change was observed after manipulation, demonstrating that the biofilm was not noticeably degraded by the immunofluorescence staining procedure (data not shown).

### Purification of RbmA-FLAG and Bap1-FLAG

Wild-type *V. cholerae* carrying either a RbmA-FLAG or a Bap1-FLAG expression plasmid were grown overnight on an LB agar plate containing ampicillin. Several of the resulting colonies were inoculated into 100 mls of LB broth supplemented with ampicillin. When the culture reached mid-log phase, IPTG was added to a final concentration of 1 mM. After 4 hours of additional growth, the cells were pelleted at 5,000 rpm at 4°C (Sorvall, rotor SLA-600TC), and the recovered supernatant was distributed into two 50 ml conical tubes. 200 µl of Anti-FLAG M2 Affinity Gel prepared according to the manufacturer's instructions (Sigma-Aldrich) was added to each tube, and the tubes were agitated for 1 hour at room temperature to allow the protein to adhere to the resin. The resin was collected in 10 ml chromatography columns (Bio-Rad) and washed with 2×10 ml PBS. Proteins bound to the resin were eluted with 300 µl of 0.1 M glycine, pH 2.5 and instantly brought to pH 8 by addition of 10 mM Tris, pH 8. Protein concentration was determined by absorbance at 280 nm, and the eluate was analysed by SDS-PAGE using a 12% pre-cast gel (Pierce). After separation, the gel was stained with Imperial Stain (Pierce).

### *V. cholerae* biofilm co-culture experiments

For quantification, equal numbers of *lacZ^+^* and *lacZ^−^ V. cholerae* strains were inoculated into LB-filled wells of a microtiter dish, and biofilms were allowed to form at 27°C over 24 hours. Biofilms were then disrupted with 1 mm glass beads, and serial dilutions of the resulting cell suspensions were plated for isolation on LB agar plates containing X-GAL. In the morning, numbers of blue and white colonies were recorded.

For microscopy, equal numbers of a Δ*rbmC*Δ*bap1* mutant and a Δ*vpsL* mutant carrying a chromosomally-encoded, constitutively expressed *gfp* allele and a plasmid-encoded *bap1*-FLAG allele were inoculated into LB-filled wells of a microtiter dish with a coverslip. Biofilms were allowed to form as described above. Biofilms formed on coverslips were subsequently removed and prepared for immunofluorescence as as described above. These biofilms were examined by confocal microscopy using the LSM700 microscope (Zeiss).

### Evaluation of monolayer formation

Cells were grown in a 24 well microtiter dish filled with minimal medium (MM) alone or supplemented with purified Bap1 or BSA. An Eclipse TE-2000-E phase contrast microscope (Nikon) equipped with a 20X objective and an Orca digital CCD camera (Hamamatsu) was used to obtain images. Surface area coverage was calculated using IP Lab software (Nikon). Two randomly selected fields were measured in each of three biological replicates.

### Accession numbers

Proteins listed in Tables 1 and 2 have the following Swiss Prot accession numbers. Table 1: MshA (Q60074), RbmA (Q9KTH4), RbmC (Q9KTH2), FlaB (P0C6C4), FlaD (P0C6C6), FlaC (P0C6C5), FlaA (P0C6C3), ChiA-2 (Q9KND8), HlyA (P09545), and HAP (P24153). Table 2: VC0174 (Q9KVH2), VC0430 (Q9KUT5), VC0483 (Q9KUN2), VC1101 (Q9KT04), VC1154 (Q9KSV2), VC1334 (Q9KSC4), VC1384 (Q9KS75), VC1523 (Q9KRW1), VC1834 (Q9KR13), VC1853 (Q9KQZ4), VC1887 (Q9KQW1), VC1894 (Q9KQV4), VC2168 (Q9KQ36), VC2517 (Q9KP59), VCA0026 (Y2826), VCA0058 (Q9KNA7), VCA0144 (Q9KN22), and VCA0900 (Q9KL48).

## Supporting Information

Figure S1 **Overview of proteins derived from proteomic analysis of the biofilm matrix.** Percentage and absolute number of cytoplasmic and extracytoplasmic proteins derived from each method of biofilm matrix protein purification. (i) Vortexing in the presence of beads, (ii) biotinylation, vortexing in the presence of beads, and purification with streptavidin, (iii) biotinylation, sonication, and purification with streptavidin, and (iv) biotinylation, vortexing without beads, and purification with streptavidin. More gentle methods of biofilm disruption yielded a greater percentage of extracellular proteins but a smaller absolute number of proteins.
(TIF)

Figure S2 **Bap1 and RbmC perform redundant functions in biofilm formation.** Quantification of biofilms formed by wild-type *V. cholerae*, a Δ*bap1*Δ*rbmC* mutant alone, or a Δ*bap1*Δ*rbmC* mutant rescued with a pBAD plasmid carrying a wild-type *bap1* allele, a wild-type *rbmC* allele, or *rbmC*-C140 truncated allele. * indicates values that are statistically significantly different from wild-type.
(TIF)

Table S1 **Proteins identified in the proteomic analyses.**
(DOC)

Table S2 **Strains and plasmids.**
(DOC)
