# Supplementary figures and images for: A Communal Bacterial Adhesin Anchors Biofilm and Bystander Cells to Surfaces
Source: PLoS Pathog. 2011 Aug 25;7(8):e1002210. doi: 10.1371/journal.ppat.1002210 (PMC3161981; doi:10.1371/journal.ppat.1002210)

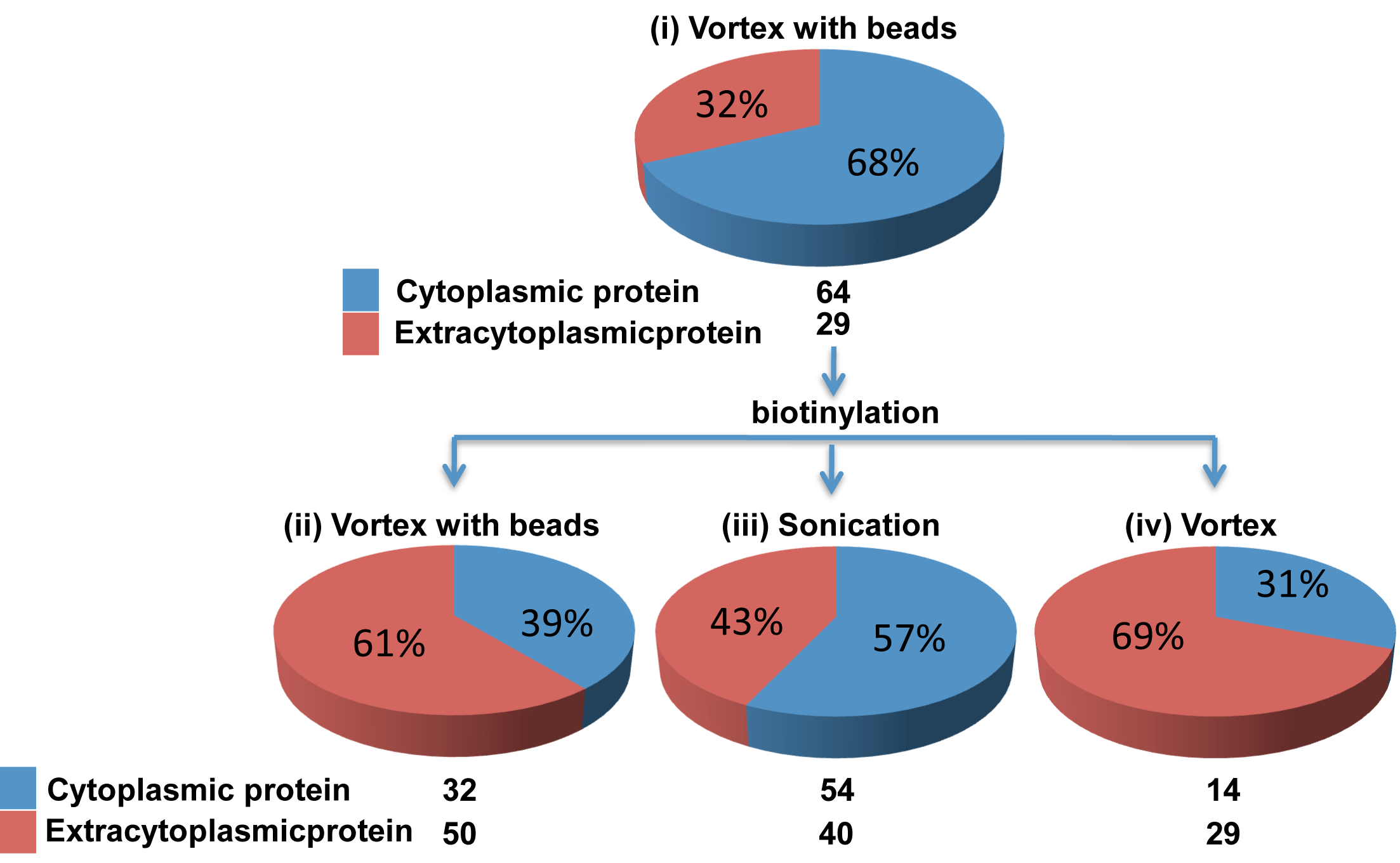

Supplement: Figure S1 — Overview of proteins derived from proteomic analysis of the biofilm matrix. Percentage and absolute number of cytoplasmic and extracytoplasmic proteins derived from each method of biofilm matrix protein purification. (i) Vortexing in the presence of beads, (ii) biotinylation, vortexing in the presence of beads, and purification with streptavidin, (iii) biotinylation, sonication, and purification with streptavidin, and (iv) biotinylation, vortexing without beads, and purification with streptavidin. More gentle methods of biofilm disruption yielded a greater percentage of extracellular proteins but a smaller absolute number of proteins. (TIF) [file ppat.1002210.s001.tif]

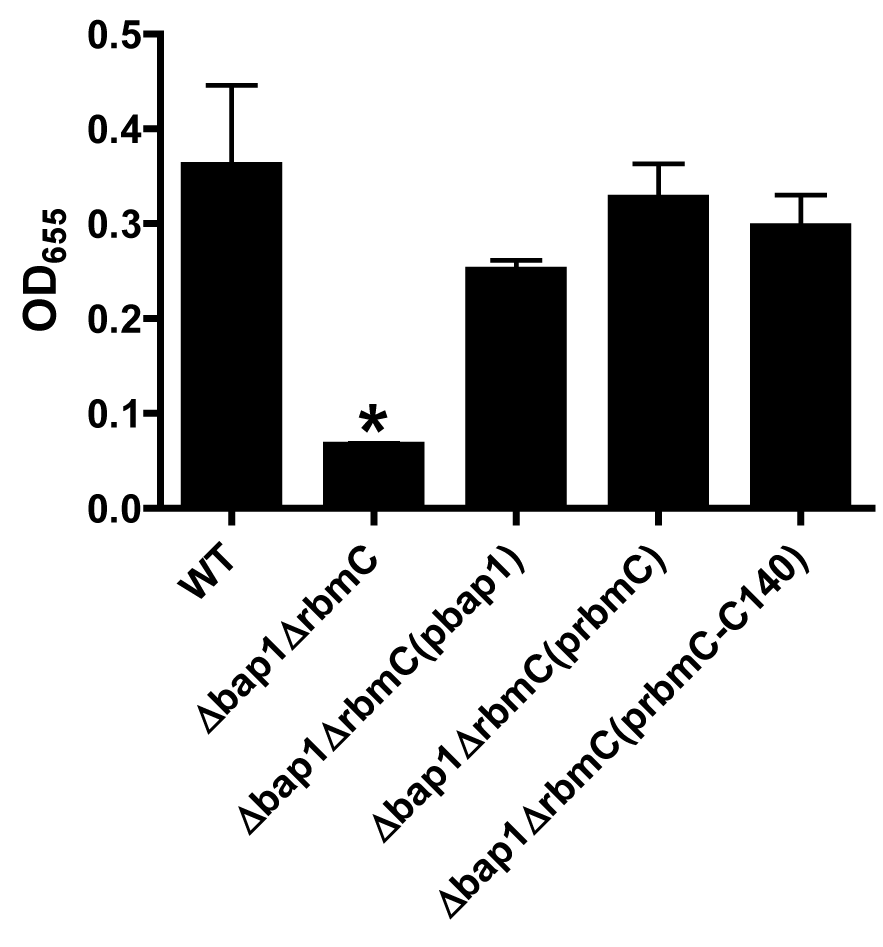

Supplement: Figure S2 — Bap1 and RbmC perform redundant functions in biofilm formation. Quantification of biofilms formed by wild-type V. cholerae, a Δbap1ΔrbmC mutant alone, or a Δbap1ΔrbmC mutant rescued with a pBAD plasmid carrying a wild-type bap1 allele, a wild-type rbmC allele, or rbmC-C140 truncated allele. * indicates values that are statistically significantly different from wild-type. (TIF) [file ppat.1002210.s002.tif]
