# Supplementary material for: A Communal Bacterial Adhesin Anchors Biofilm and Bystander Cells to Surfaces
Source: PLoS Pathog. 2011 Aug 25;7(8):e1002210. doi: 10.1371/journal.ppat.1002210 (PMC3161981; doi:10.1371/journal.ppat.1002210)
Supplement: Table S1 — Proteins identified in the proteomic analyses. (DOC) [file ppat.1002210.s003.doc]

| **Supplementary Table 1: Proteins identified in proteomic analysis** | | |  |
| --- | --- | --- | --- |
| **Locus** | **Annotation** | **Program used to predict secretion** | **Purification methods in which protein was identified*** |
|  |  |  |  |
| VC0409 | MshA | Fimbria | i;ii |
|  |  |  |  |
| VC1064 | lipoprotein-related protein | LIPOP | i |
| VC1162 | lipoprotein, putative | LIPOP | iv |
| VC1663 | HslJ | LIPOP | ii;iv |
| VC1835 | peptidoglycan-associated lipoprotein | LIPOP | ii;iv |
| VC1894 | hypothetical protein | LIPOP | ii |
| VC2174 | UshA | LIPOP | ii;iii;iv |
| VCA1027 | maltose operon periplasmic protein, putative | LIPOP | i;ii;iv |
|  |  |  |  |
| VC1987 | outer membrane lipoprotein Slp, putative | SECRETOMEP | ii;iv |
| VC2045 | superoxide dismutase, Fe | SECRETOMEP | i;ii;iii;iv |
| VC2142 | FlaB | SECRETOMEP | ii;iii |
| VC2143 | FlaD | SECRETOMEP | i;ii;iii |
| VC2187 | FlaC | SECRETOMEP | i;ii |
| VC2188 | FlaA | SECRETOMEP | ii |
| VC2562 | CpdB | SECRETOMEP | i;iii |
|  |  |  |  |
| VC0010 | amino acid ABC transporter, periplasmic amino | SIGNALP | ii;iii |
| VC0034 | TcpG | SIGNALP | iii |
| VC0168 | cytochrome c5 | SIGNALP | ii;iii |
| VC0171 | peptide ABC transporter, periplasmic peptide-binding | SIGNALP | i;ii;iii;iv |
| VC0174 | hypothetical protein | SIGNALP | iii |
| VC0194 | gamma-glutamyltranspeptidase | SIGNALP | iii |
| VC0430 | immunogenic protein | SIGNALP | i;ii;iii |
| VC0445 | SurA | SIGNALP | iv |
| VC0483 | hypothetical protein | SIGNALP | iii |
| VC0566 | protease DO | SIGNALP | ii;iii |
| VC0608 | iron(III) ABC transporter, periplasmic | SIGNALP | i;ii;iii;iv |
| VC0633 | outer membrane protein OmpU | SIGNALP | i;ii;iii;iv |
| VC0642 | N utilization substance protein A | SIGNALP | i |
| VC0928 | RbmA | SIGNALP | i;ii |
| VC0930 | RbmC | SIGNALP | iii |
| VC1043 | long-chain fatty acid transport protein | SIGNALP | i;ii;iv |
| VC1091 | oligopeptide ABC transporter, periplasmic | SIGNALP | i;ii;iii;iv |
| VC1101 | hypothetical protein | SIGNALP | ii;iii |
| VC1154 | hypothetical protein | SIGNALP | ii |
| VC1288 | periplasmic glucans biosynthesis protein MdoG | SIGNALP | iii |
| VC1334 | hypothetical protein | SIGNALP | i;ii;iii |
| VC1362 | amino acid ABC transporter, periplasmic amino | SIGNALP | i;ii;iii |
| VC1384 | hypothetical protein | SIGNALP | ii |
| VC1424 | spermidine/putrescine ABC transporter, periplasmic | SIGNALP | ii |
| VC1425 | spermidine/putrescine ABC transporter, periplasmic | SIGNALP | ii |
| VC1496 | carboxy-terminal protease | SIGNALP | iv |
| VC1523 | hypothetical protein | SIGNALP | ii;iii |
| VC1621 | agglutination protein | SIGNALP | ii;iv |
| VC1834 | hypothetical protein | SIGNALP | ii;iv |
| VC1836 | TolB | SIGNALP | ii;iii;iv |
| VC1853 | hypothetical protein | SIGNALP | iii |
| VC1854 | OmpT protein | SIGNALP | ii |
| VC1863 | amino acid ABC transporter, periplasmic amino | SIGNALP | i;ii;iii;iv |
| VC1887 | hypothetical protein | SIGNALP | ii;iv |
| VC1929 | C4-dicarboxylate-binding periplasmic protein | SIGNALP | i;ii;iii |
| VC2168 | hypothetical protein | SIGNALP | i;ii;iv |
| VC2213 | outer membrane protein OmpA | SIGNALP | i;ii;iii;iv |
| VC2251 | outer membrane protein OmpH | SIGNALP | ii;iii;iv |
| VC2305 | outer membrane protein OmpK | SIGNALP | i |
| VC2436 | outer membrane protein TolC | SIGNALP | ii;iv |
| VCA0026 | hypothetical protein | SIGNALP | iii;iv |
| VCA0027 | chitinase | SIGNALP | i |
| VCA0058 | conserved hypothetical protein | SIGNALP | iv |
| VCA0130 | D-ribose transporter subunit RbsB | SIGNALP | ii;iii |
| VCA0144 | immunogenic protein | SIGNALP | iii |
| VCA0219 | Hemolysin precursor | SIGNALP | i |
| VCA0576 | heme transport protein HutA | SIGNALP | ii |
| VCA0591 | peptide ABC transporter, periplasmic peptide-binding | SIGNALP | iii |
| VCA0685 | iron(III) ABC transporter, periplasmic | SIGNALP | ii |
| VCA0759 | arginine ABC transporter, periplasmic | SIGNALP | ii;iii |
| VCA0865 | Hemagglutinin/proteinase precursor (HA/protease) (Vibriolysin) | SIGNALP | i |
| VCA0867 | outer membrane protein W | SIGNALP | ii |
| VCA0900 | hypothetical protein | SIGNALP | iii |
| VCA0945 | maltose ABC transporter, periplasmic maltose-binding protein (malE) | SIGNALP | i;ii;iii;iv |
| VCA1028 | maltoporin (ompS) | SIGNALP | i;ii;iii;iv |
|  |  |  |  |
| VC2092 | citrate synthase (gltA) [2.3.3.1] | TMHMM | i;ii;iii |
| VC2517 | hypothetical protein | TMHMM | iii;iv |
| VCA0070 | phosphate ABC transporter, periplasmic | TMHMM | i;iv |
| VCA0139 | hypothetical protein | TMHMM | iii |

* Refers biofilm matrix preparation in which protein was identified as follows: (i) Biofilm matrix was removed by vortexing of biofilm matrix cells with beads. (ii) Extracytoplasmic proteins were biotinylated prior to removal of biofilm matrix by vortexing of biofilm matrix cells with beads. (iii) Extracytoplasmic proteins were biotinylated prior to disruption of the biofilm by sonication. (iv) Extracytoplasmic proteins were biotinylated prior to removal of biofilm matrix by vortexing of biofilm matrix cells. In all preparations where biotinylation was performed, a Neutravidin column was used to enrich for extracytoplasmic proteins.
