# Supplementary material for: A Communal Bacterial Adhesin Anchors Biofilm and Bystander Cells to Surfaces
Source: PLoS Pathog. 2011 Aug 25;7(8):e1002210. doi: 10.1371/journal.ppat.1002210 (PMC3161981; doi:10.1371/journal.ppat.1002210)
Supplement: Table S2 — Strains and plasmids. (DOC) [file ppat.1002210.s004.doc]

**Table S2: Strains and plasmids**

| **Bacterial Strains** | **Genotype and/or phenotype** | **Reference** |
| --- | --- | --- |
|  |  |  |
| *E. coli strains* |  |  |
| SM10λpir | *thi thr leu tonA lacY supE recA::RP4-2-Tc::MuλpirR6K*;Km^r^ | [1] |
| *V. cholerae* strains |  |  |
| PW249 | MO10 ; Sm^r^ | [2] |
| PW328 | MO10 Δ*vpsL,* Sm^r^ | [3] |
| PW357 | MO10 *lacZ*::*vpsLp*→*lacZ*; Sm^r^ | [3] |
| PW454 | MO10 Δ*vpsL lacZ::gfp*; Sm^r^ | This study |
| PW707 | MO10 Δ*bap1*Δ*rbmC ; lacZ*::*vpsLp*→*lacZ ;* Sm^r^ | This study |
| PW1085 | MO10 *rbmA-flag*;  *lacZ*::*vpsLp*→*lacZ ;*Sm^r^ Ap^r^ | This study |
| PW1086 | MO10 *bap1-flag*;  *lacZ*::*vpsLp*→*lacZ ;* Sm^r^ Ap^r^ | This study |
| PW1087 | MO10 Δ*rbmA ; lacZ*::*vpsLp*→*lacZ ;* Sm^r^ | This study |
| PW1088-90 | MO10 Δ*bap1*Δ*rbmC*, Sm^r^ | This study |
|  |  |  |
| **Plasmids** |  |  |
|  |  |  |
| pWM91 | *oriR6KmobRP4 lacI pTac tnp miniTn10Km*; Ap^r^ | [4] |
| pWM91Δ*bap1* | pWM91 carrying a fragment of *bap1* harboring an internal, unmarked deletion; Ap^r^ | [5] |
| pWM91Δ*rbmC* | pWM91 carrying a fragment of *rbmC* harboring an internal, unmarked deletion; Ap^r^ | This study |
| pWM91Δ*rbmA* | pWM91 carrying a fragment of *rbmA* harboring an internal, unmarked deletion; Ap^r^ | This study |
| pGP704::*bap1-flag* | pGP704 carrying 3’ end of *bap1* fused to a *flag* tag; Ap^r^ | This study |
| pGP704::*rbmA-flag* | pGP704 carrying 3’ end of *rbmA* fused to a *flag* tag; Ap^r^ | This study |
| pJZ111 | Plac::gfp::lacZ in pCVD442; Ap^r^ | [6] |
| pBAD-TOPO-*rbmA* | pBAD-TOPO carrying the gene at locus VC0928 (*rbmA*); Ap^r^ | This study |
| pFLAG-*chiA-2* | pFLAG-CTC carrying the gene at locus VCA0027 (*chiA-2*); Ap^r^ | This study |
| pFLAG-*hlyA* | pFLAG-CTC carrying the gene at locus VCA0219 (*hlyA*); Ap^r^ | This study |
| pFLAG-*hapA* | pFLAG-CTC carrying the gene at locus VCA0865 (*hapA*); Ap^r^ | This study |
| pFLAG-*tcpG* | pFLAG-CTC carrying the gene at locus VC0034 *(tcpG*); Ap^r^ | This study |
| pFLAG-*mshA* | pFLAG-CTC carrying the gene at locus VC0409 (*mshA*); Ap^r^ | This study |
| pFLAG-*rbmA* | pFLAG-CTC carrying the gene at locus VC0928 (*rbmA*); Ap^r^ | This study |
| pFLAG-*crr* | pFLAG-CTC carrying the gene at locus VC0964 (*crr*); Ap^r^ | This study |
| pFLAG-*bap1* | pFLAG-CTC carrying the gene at locus VC1888 (*bap1*); Ap^r^ | This study |

1. Miller VL, Mekalanos JJ (1988) A novel suicide vector and its use in construction of insertion mutations: osmoregulation of outer membrane proteins and virulence determinants in *Vibrio cholerae* requires *toxR*. J Bacteriol 170: 2575-2583.

2. Waldor MK, Colwell R, Mekalanos JJ (1994) The *Vibrio cholerae* O139 serogroup antigen includes an O-polysaccharide capsule and lipopolysaccharide virulence determinant. Proc Natl Acad Sci USA 91: 11388-11392.

3. Haugo AJ, Watnick PI (2002) *Vibrio cholerae* CytR is a repressor of biofilm development. Mol Microbiol 45: 471-483.

4. Metcalf WW, Jiang W, Daniels LL, Kim SK, Haldimann A, et al. (1996) Conditionally replicative and conjugative plasmids carrying *lacZ* a for cloning, mutagenesis, and allele replacement in bacteria. Plasmid 35: 1-13.

5. Moorthy S, Watnick PI (2005) Identification of novel stage-specific genetic requirements through whole genome transcription profiling of Vibrio cholerae biofilm development. Mol Microbiol 57: 1623-1635.

6. Bomchil N, Watnick P, Kolter R (2003) Identification and characterization of a Vibrio cholerae gene, mbaA, involved in maintenance of biofilm architecture. J Bacteriol 185: 1384-1390.
